# Supplementary figures and images for: The invasive pathogen Yersinia pestis disrupts host blood vasculature to spread and provoke hemorrhages
Source: PLoS Negl Trop Dis. 2021 Oct 5;15(10):e0009832. doi: 10.1371/journal.pntd.0009832 (PMC8519436; doi:10.1371/journal.pntd.0009832)

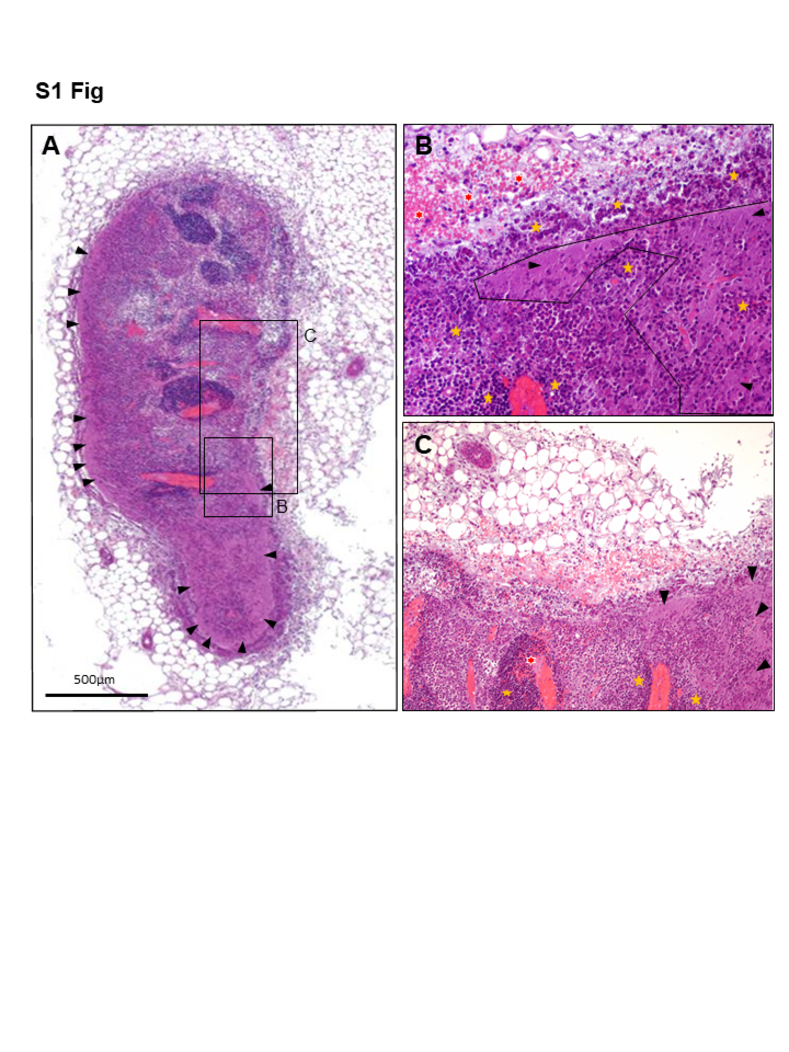

Supplement: S1 Fig — Histological section of a mouse inguinal lymph node (4 μm thick) stained with hematoxylin-eosin. ORF1 mouse was infected subcutaneously with 500cfu CO92 Y. pestis strain for 76h prior to sacrifice. A. Full section of the lymph node. Large infiltrate of Y. pestis, colored in pink, are visible at the periphery of the lymph node (black arrowheads), progressing from the lymphatic sinus to the cortex. Edemas and large engorged blood vessels (erythrocytes are colored in red) are visible within the lymph node. Infiltrate of Polynuclear neutrophils (PMN) with the characteristic horseshoe shaped nuclei colored in blue/purple are visible in the cortex. Bar = 500 μm B. Higher magnification of the square B displaying a “sea” of bacteria (black arrowheads, black frame surrounding pink areas) and PMNs infiltrates around the bacteria (yellow five-branch stars). Hemorrhages are visible in the tissue surrounding the lymph node (six-branch red stars). C. Higher magnification of the square C displaying a “sea” of bacteria (black arrowheads), PMNs infiltrates (yellow five-branch stars) and enlarged engorged blood vessels (red). A hemorrhage is visible in the cortex of the lymph node (six-branch red stars). Bacteria are visible are close proximity of the blood vessel. Blood vessels are tampered, but not fully degraded. (TIF) [file pntd.0009832.s005.tif]

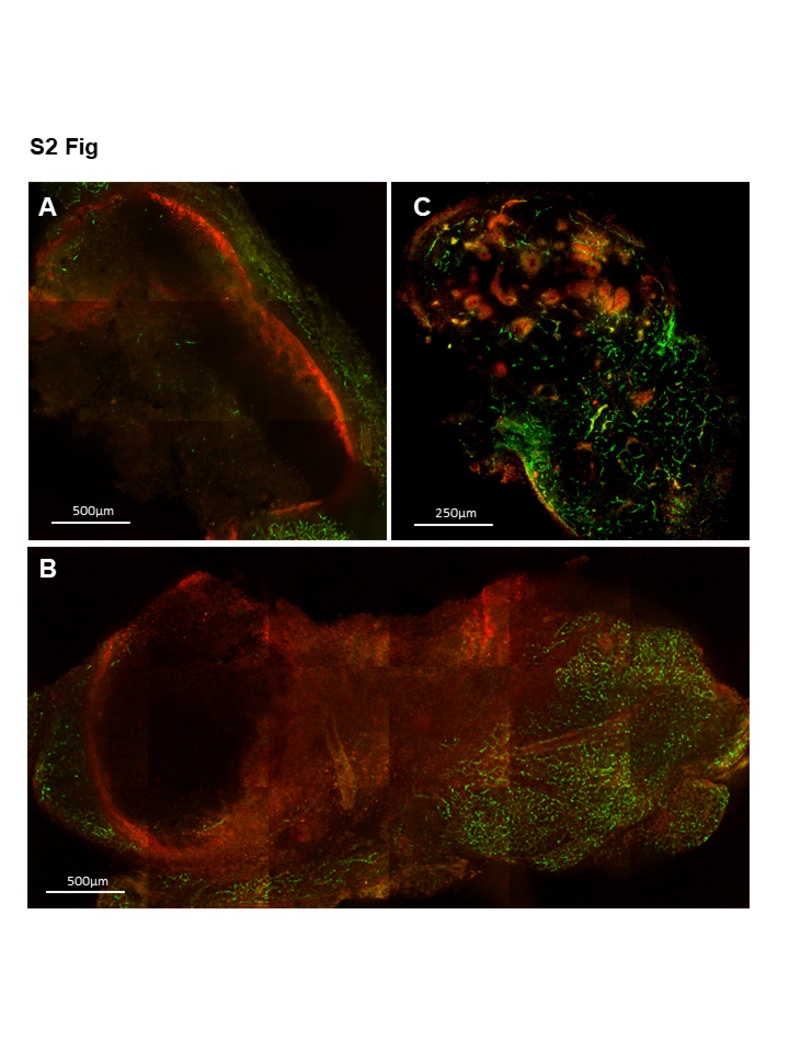

Supplement: S2 Fig — Inguinal draining lymph nodes and secondary lymph node of Y. pestis-infected mice displaying an advanced state of degradation on D3 post-infection. The images are reconstructed from panels corresponding to the maximum of intensity (MIP) calculated on 150–200 um sections. Red and green panels are merged. A. and B. are two consecutive slices of the same draining lymph node. Red bacteria are filling the entire volume of the cortex, all vasculature within the cortex seems destroyed. C. is a secondary lymph node infected through blood circulation. Dense spots of bacteria spread through the cortex from the initial entry point. Blood vessels within the cortex have progressively disappeared on the side of numerous bacterial areas (top half) compared to the other side (bottom half). (TIF) [file pntd.0009832.s006.tif]

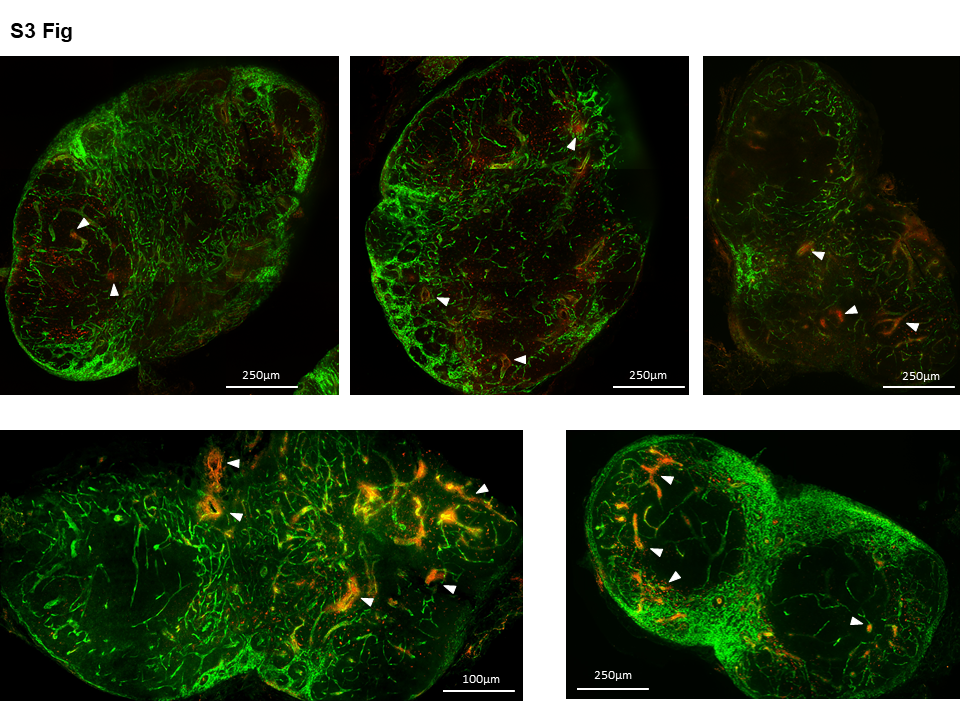

Supplement: S3 Fig — Secondary lymph nodes of 5 Y. pestis-infected mice on D3 post-infection. The images are reconstructed from panels corresponding to the maximum of intensity (MIP) calculated on 150–200 μm sections. Bacteria are tagged with RFP and appeared as red spots co-localizing with GFP-tagged blood vessels (arrowheads). Red and green panels are merged. The average size of a lymph node is 1 to 2 mm. (TIF) [file pntd.0009832.s007.tif]

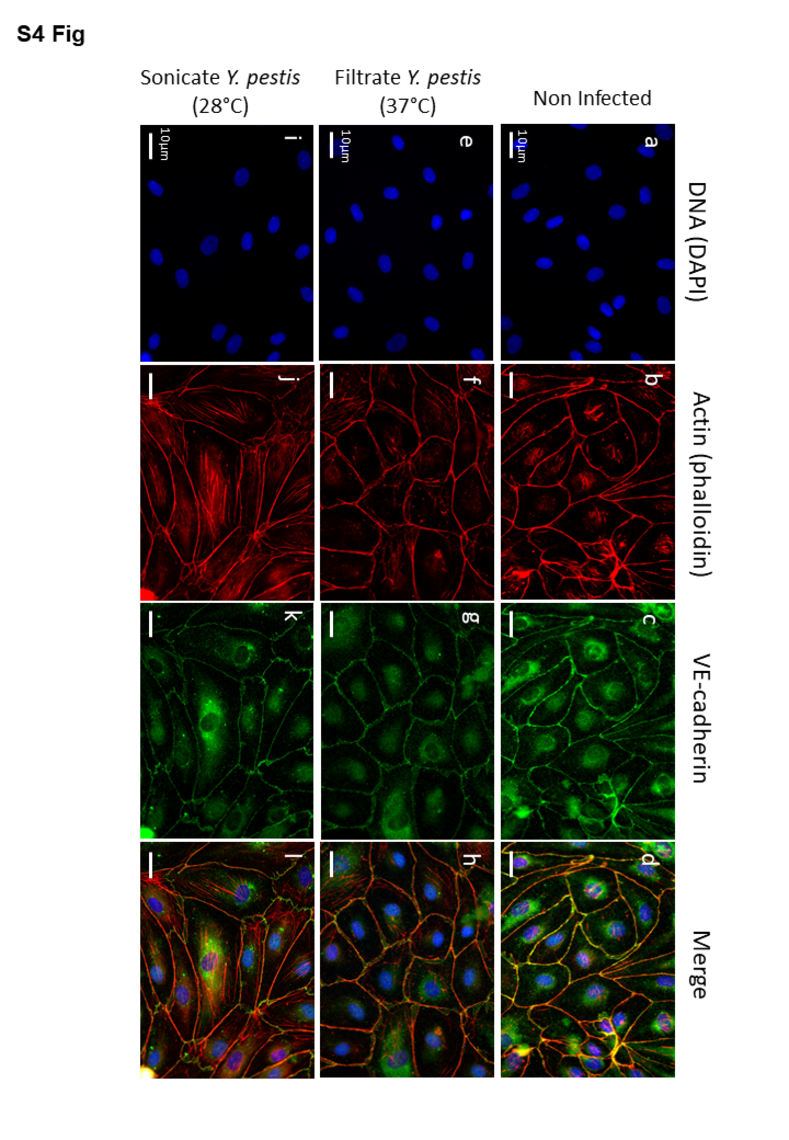

Supplement: S4 Fig — HDMEC monolayers were incubated for 5h with the equivalent of 107 bacteria Y. pestis culture medium (filtrate) or 107 bacteria sonicate (see Materials and Methods section). No holes in the tight junctions are observed due to these treatments. DNA was stained with DAPI (blue; panels a,e,i). Actin was stained with Phalloidin (Red; panels b,f,j). Tight junctions were targeted with an anti-VE-cadherin antibody (green, panels c,g,k).(d,h,l) are merged panels. Bar = 10 μm. (TIF) [file pntd.0009832.s008.tif]

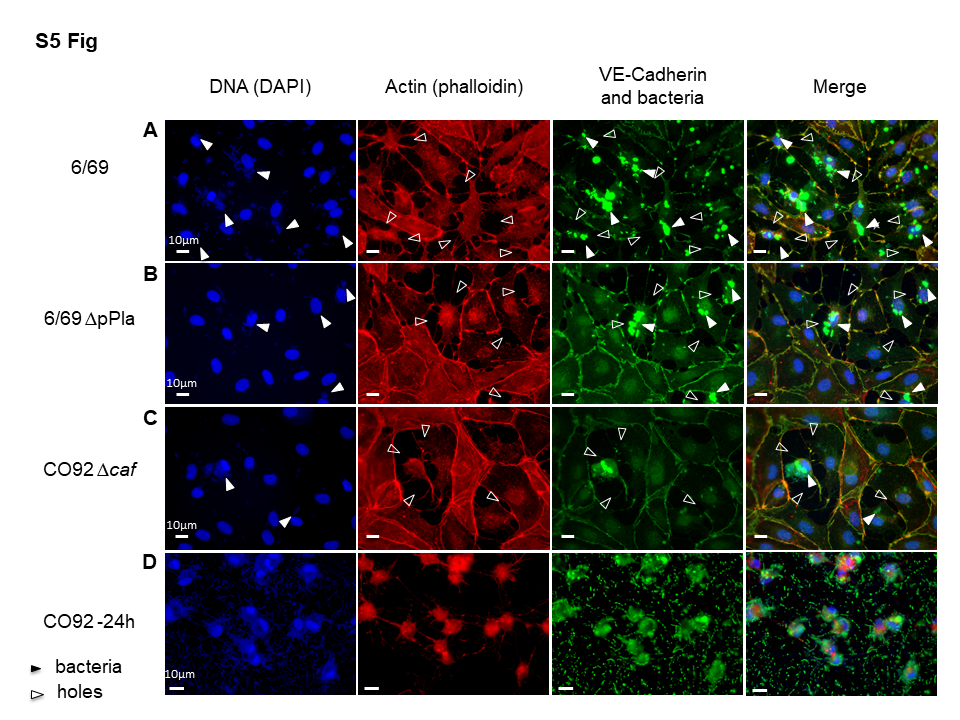

Supplement: S5 Fig — HDMEC monolayers were infected (MOI = 100) for 2.5h with Y. pestis 6/69 wild type (A), its derivative cured of pPla (B), and Y. pestis CO92 Δcaf (C). D. HDMEC monolayers were infected (MOI = 100) for 24h with Y. pestis CO92. DNA was stained with DAPI (blue; panels a,e,i,m). Actin was stained with Phalloidin (Red; panels b,f,j,n). Staining was done as described in the legend of Fig 4. Examples of bacteria in close contact with the cells are indicated with white plain arrowheads. Bar = 10 μm. (TIF) [file pntd.0009832.s009.tif]
