# Supplementary material for: The invasive pathogen Yersinia pestis disrupts host blood vasculature to spread and provoke hemorrhages
Source: PLoS Negl Trop Dis. 2021 Oct 5;15(10):e0009832. doi: 10.1371/journal.pntd.0009832 (PMC8519436; doi:10.1371/journal.pntd.0009832)
Supplement: Attachment — Submitted filename: Reviewer_responses.docx [file pntd.0009832.s010.docx]

Dear Dr. Mikaty,
 
Thank you very much for submitting your manuscript "The invasive pathogen   Yersinia pestis   disrupts host blood vasculature to spread and provoke hemorrhages." for consideration at PLOS Neglected Tropical Diseases. As with all papers reviewed by the journal, your manuscript was reviewed by members of the editorial board and by several independent reviewers. In light of the reviews (below this email), we would like to invite the resubmission of a significantly-revised version that takes into account the reviewers' comments.
 
I have received the reviews for your manuscript. The reviewers have evaluated your manuscript and find it valuable to the field. However, they raise a few important questions. Additional data is needed to resolve them. A fully revised manuscript will be evaluated before publication in PLOS NTD.

 We cannot make any decision about publication until we have seen the revised manuscript and your response to the reviewers' comments. Your revised manuscript is also likely to be sent to reviewers for further evaluation.
 
When you are ready to resubmit, please upload the following:

[1] A letter containing a detailed list of your responses to the review comments and a description of the changes you have made in the manuscript. Please note while forming your response, if your article is accepted, you may have the opportunity to make the peer review history publicly available. The record will include editor decision letters (with reviews) and your responses to reviewer comments. If eligible, we will contact you to opt in or out.

[2] Two versions of the revised manuscript: one with either highlights or tracked changes denoting where the text has been changed; the other a clean version (uploaded as the manuscript file).

Important additional instructions are given below your reviewer comments.

Please prepare and submit your revised manuscript within 60 days. If you anticipate any delay, please let us know the expected resubmission date by replying to this email. Please note that revised manuscripts received after the 60-day due date may require evaluation and peer review similar to newly submitted manuscripts.
 
Thank you again for your submission. We hope that our editorial process has been constructive so far, and we welcome your feedback at any time. Please don't hesitate to contact us if you have any questions or comments.
 
Sincerely,
R. Manjunatha Kini
Associate Editor
PLOS Neglected Tropical Diseases

Fabiano Oliveira
Deputy Editor
PLOS Neglected Tropical Diseases

Dear Dr. Manjunatha Kini, dear Dr. Fabiano Oliveira,

Thank you for your consideration of our manuscript "The invasive pathogen *Yersinia pestis* disrupts host blood vasculature to spread and provoke hemorrhages." for publication in PLOS Neglected Tropical Diseases. We are glad our work was so well received.

We thank the reviewers for their appreciation and fruitful comments.

The major changes to the article are the following: (i) we changed supplementary figure Fig S1 to a core figure (now Fig 2), as proposed by reviewer #1 and #2; (ii) some results were added as supplementary figures (now Fig S1 and S2) to answer some questions or details asked by the reviewers; (iii) a discussion on the inflammatory process during the invasion of *Y. pestis* and how it would affect blood vessels integrity has been added as requested by reviewer #1 and #3.

The new Fig S1 is a histopathology picture of a draining lymph node (dLN) at D3 post-infection, at a level of infection close to the one in Fig 1. It shows the inflammatory process during *Y. pestis* invasion, notably the recruitment of PMNs, but also the integrity of blood vessels that is quite conserved despite the inflammatory process, contrary to blood vessels that are altered in the middle of the bacterial mass. This figure also helps to better apprehend Fig 1, as it shows the same phenomenon with a different perspective. L. Fiette, who did the histological analysis, has been added to the author list.

The new Fig S2 presents a draining LN and a secondary LN at a more advanced stage of infection. We believe that a later time point is actually more informative than an earlier time point as requested by reviewer #1 and answers better her/his comments. These figures show the very characteristic features of the degradation of the LN depending on the entry door (lymphatic or blood) and show indisputably that Fig 1C corresponds to an infection through the lymphatic door and not through blood.

The revised version of the manuscript with tracks displays the tracking of all changes. Modifications corresponding to change in response to the reviewer are also highlighted in yellow.

We could not answer reviewers #1 and #3 request for additional experiments to decipher the molecular basis of the phenomenon regarding the Type Three Secretion System (TTSS) and the Yop effectors involved. The experiments proposed are of interest, and are part of another ongoing study attempting to identify the bacterial factors involved in the peculiar histopathological features observed. This will need numerous additional experiments that will go beyond the message of this work, which is to demonstrate the previously undocumented degradation of blood vessels by *Y. pestis,* with a direct contact between bacteria and the endothelium in the lymph node.

We hope this revised version of our manuscript and our answers to the reviewers' comments will make this manuscript suitable for publication in PLOS Neglected Tropical Diseases.

Sincerely,

Guillain MIKATY.

_____________________________________________________________________________________________________________

Reviewer's Responses to Questions

**Key Review Criteria Required for Acceptance?**
As you describe the new analyses required for acceptance, please consider the following:</br></br>

**Methods**
-Are the objectives of the study clearly articulated with a clear testable hypothesis stated?</br>
-Is the study design appropriate to address the stated objectives?</br>
-Is the population clearly described and appropriate for the hypothesis being tested?</br>
-Is the sample size sufficient to ensure adequate power to address the hypothesis being tested?</br>
-Were correct statistical analysis used to support conclusions?</br>
-Are there concerns about ethical or regulatory requirements being met?</br></br>

**Reviewer #1**: See my comments below

**Reviewer #2**: Methods were appropriate to ask the relevant questions.

**Reviewer #3**: Methods are appropriately described.

Reviewer 1 comment has been addressed in the corresponding section.

_____________________________________________________________________________________________________________


**Results**

-Does the analysis presented match the analysis plan?</br>
-Are the results clearly and completely presented?</br>
-Are the figures (Tables, Images) of sufficient quality for clarity?</br></br>

**Reviewer #1**: See my comments below

**Reviewer #2**: The results are presented clearly and concisely, and Figures are of high quality.

**Reviewer #3**: Results are clearly described.

Reviewer 1 comment has been addressed in the corresponding section.

_____________________________________________________________________________________________________________


**Conclusions**
-Are the conclusions supported by the data presented?</br>
-Are the limitations of analysis clearly described?</br>
-Do the authors discuss how these data can be helpful to advance our understanding of the topic under study?</br>
-Is public health relevance addressed?</br></br>

**Reviewer #1**: **Major Concerns:**

1)      The authors’ main conclusion in this paper is that Y. pestis, via a T3SS-dependent mechanism, actively damages the blood vessel endothelium, thus increasing vessel permeability, and this is how the bacteria enter the bloodstream and disseminate.  I do not feel that the data the authors present here allow them to rule out the other 2 potential mechanisms of dissemination to the blood described by the authors in the introduction (movement of bacteria through the lymph and eventually into bloodstream, or carriage of bacteria into the blood by phagocytes).  I can see how damage to the vessels would cause leakage out of the vessels, but that does not necessarily mean bacteria would enter those damaged vessels.

The reviewer is right, the data presented cannot rule out the two other hypotheses proposed regarding the pathway used by *Y. pestis* to reach the bloodstream (in addition to a rare but possible direct injection by the fleabite responsible for a quick septicemic plague). Previous work by Nahm and colleagues (Nahm et al., PLoS ONE, 2012) showed that the variable timing between the subcutaneous infection of *Y. pestis* and the outcome is due to the time to reach the draining Lymph Node (dLN). Once in the dLN, *Y. pestis* quickly spreads to other organs, the infection is rapid and often fatal, suggesting that LN are the entry door to blood circulation. The main argument against the hypothesis for a direct entry of the bacillus into the bloodstream was the absence of proof that *Y. pestis* enters in contact with vessels and could cross the vascular barrier. Our work demonstrates that both events take place *in vivo*, thus answering the critics of the hypothesis. Though it does not invalidate other hypotheses, we believe our work strongly supports a direct entry of *Y. pestis.* The text has been amended to clarify and moderate this point according to the reviewer’s comment (l299, would -> could; l455-465: text added)

2)      At the time point chosen by the authors for their in vivo experiments, there are really two different phenomena being studied regarding Y. pestis dissemination and it is a bit difficult to separate the two.  The first relates to how the bacteria are entering the bloodstream.  The second relates to how these disseminated bacteria move from the blood into the tissue parenchyma.  For the first, since the bacteria would initially be found in the subcapsular sinus area early in infection, the bacteria would need to gain access to the LN parenchyma to come in direct contact with blood vessels to increase their permeability.  The authors do not address how the bacteria might be getting to the parenchyma initially.  Because of the later time point (d3), the blood vessel damage the authors see in the dLN could have been caused from the inside out by the bacteria already spreading systemically, rather than the bacteria that arrived in the LN via afferent lymph from the injection site.  For the second, it’s easier to see how the bacteria already in the bloodstream could damage the vessels and leak out into the parenchyma and cause the hemorrhages the authors discuss.  I feel that this study would benefit from the addition of earlier time points for the in vivo work.  Being able to show the early stages of blood vessel damage, or at least bacteria in contact with vessels and able to inject Type III effectors, prior to dissemination of the bacteria throughout the body, would greatly strengthen the support for the authors’ conclusions.

The progression of *Y. pestis* in the proximal dLN has been described previously using histopathological analyses in different studies in mice and rats (Sebbane et al., 2005; Guinet et al., 2008). However, the mechanisms used by the bacteria to exit the lymph vasculature and reach the parenchyma are currently not known. Since our approach could not bring new insights on these mechanisms, we did not focus on this aspect of pathogenesis. The text has been amended in the introduction to shortly detail the previously published results (l65-67).

The possibility that bacteria already in the bloodstream reach the dLN at the same time they progress from the subcapsular sinus cannot be excluded. To demonstrate that the degradation of the blood vessels presented in Fig 1C is not provoked by bacteria coming from the blood, we added pictures of LN at a more advanced stage of infection, rather than an earlier time point. These pictures (Fig S2) show the characteristic outcomes of the two types of entries into the LN. The new figure presents a dLN in which the node is completely filled with bacteria, and only a few remnants of the lymph node architecture is visible (A and B), and a secondary LN infected through blood circulation at a more advanced stage (Fig S2 C). The two types of infected lymph nodes present clear different features. When infected through blood circulation, the bacterial mass stays concentrated in the shape of the vessels (new Fig 3, Fig S2C and Fig S3) and spread by diffusion. Fig 1C shows a very different pattern of bacterial localilzation around the degraded vessels. Additionally, Fig 1 shows a directed dispersion of a significant number of bacteria from the capsular sinuses to the parenchyma, characteristic of entry through the lymph door, in close proximity of the degraded vessel.

3)      The authors do not address the potential role of inflammatory cells, particularly neutrophils, could be playing in the blood vessel damage they observe.  At the later time point they focus on, there would potentially be large numbers of neutrophils recruited to the draining LN and these cells would likely colocalize with areas of high bacterial density, and therefore, areas of blood vessel damage.  The possible role of the inflammatory response in the observed phenomena should at least be discussed, if not assessed experimentally.

We thank the reviewer for this relevant point. The immune system recruitment and inhibition/degradation by *Y. pestis* might have an impact on the vascular permeability within the dLN. Abundant recruitment of macrophages and PMNs around the bacteria in the lymph node, first within the sinus, and later within the parenchyma has been described previously. The massive invasion of the parenchyma by PMNs is also visible on the newly added figure (Fig S1) that shows the histopathological analysis of a dLN on D3 post-infection, at a level of infection close to the one in Fig 1. Transmigration of PMNs through vascular barriers has been shown to provoke an increase in vascular permeability that could lead to bleeding in some cases.

However, no physical degradation of the vessels themselves, such as the one observed in the infected dLN, has been published following PMN recruitment. In the pictures presented in Fig S1, we can see a massive recruitment of PMNs around blood vessels, edemas, enlarged and engorged blood vessels, as well as focal hemorrhages. But the endothelial barrier is still visible, demonstrating that the inflammatory process itself cannot induce the destruction of the vessels as observed with *Y. pestis*. Additionally, an inflammatory process would also have a distant effect but only blood vessels in close contact with *Y. pestis* seem to be degraded. We believe the data presented exclude the possibility that the complete degradation of blood vessels observed is due to the inflammatory response. The discussion has been amended to discuss this point according to the reviewer’s comment (l444-453)

4)      For the in vitro experiments, the authors conclude that the Yops must be secreted into the HDMEC cells by the T3SS.  This is probably a reasonable assumption, but methods are available for confirming the secretion of Yops into host cell cytosol.  Determining the specific Yop or Yops responsible would also be fairly straightforward.

The reviewer is right. We think the work presented here opens for a future research project regarding the molecular determinants of the invasiveness of *Y. pestis*, the role of the TTSS, and the identification of the Yops involved in the endothelium degradation, *in vitro* and *in vivo*. This will be the basis for other studies attempting to identify the bacterial factors involved in the peculiar histopathological features observed, but this will need numerous additional experiments and will go beyond this work that forms the ground for future studies. We believe that the work presented here brings novel data and is strong and coherent enough to be published by itself.

**Reviewer #2:** The authors do a good job of contextualizing the findings and highlighting the importance to understanding bubonic plague. The conclusions are largely supported by the data. There are some inherent limitations to the microscopy, even though it is the most striking and compelling aspect of the manuscript. For instance, it is suggested that disruption of blood vessels is visualized- and that is likely the case, though it is only visual analysis and there is no conclusive evidence that it is what we are seeing (ie. control with agent that causes disruption).

The authors would like to thank the reviewer for her/his very positive comments. The reviewer points out the limitations of the *in vivo* approach developed in our study.

We considered using disruptive agents as a control (e.g. agents that would induce hemorrhages such as LPS, VEGF, or else). However, the use of these agents would cause harm to the animals, which had to be ethically justified. Furthermore, the effect on blood vessels might be too disseminated and the type of degradation of the blood vessels might probably be different from the one caused by *Y. pestis*, thus making the comparison difficult or inadequate. Other attempts to demonstrate indirectly the degradation of the blood vessels, such as observation of the autofluorescence of the collagens matrix that surrounds vessels using a bi-photon microscope, were not successful.

Eventually, the best argument that the blood vessel is indeed degraded is the 3D approach. In figure 1C, the continuity of the blood vessel is suddenly interrupted and replaced by round shapes stained in green, and the size of endothelial cells in the vicinity of bacteria. There is no more trace of the vessel in the fields scanned above and under the observed fields. Additionally, the *in vitro* approach demonstrated the possibility of the direct degradation of blood vessels by the bacteria with rounding of the cells. The text has been amended to clarify this point (l259-264).

**Reviewer #3:** The conclusions are not fully supported by the data. Some experiments are missing that are required for the conclusions made, or alternative hypotheses needed to be included.

1. It is not surprising that Y. pestis infection of endothelial cells in vitro results in cell rounding and death. There are ample reports that Y. pestis will inject effectors into almost any cell in monoculture, but cell-type specific targeting is observed in vivo that demonstrates that immune cells are preferentially targeted (independently demonstrated by Marketon, Mecsas, and Pechous labs). Therefore, these monocultures studies do not demonstrate that Y. pestis is specifically targeting endothelial cells in vivo. It is still possible that the immune response to the bacteria is leading to vascular leakage/damage in vivo that is required for immune cell recruitment, which is part of the formation of the bubo, and responsible for the localized vascular damage observed. This possibility is not properly discussed. In vivo targeting of endothelial cells in the lymph by Y. pestis using  B-lactamase reporters would provide better support for the authors’ hypothesis. Without it,
I am not sure intoxication vs. immune response hypotheses can be differentiated.

We agree with the reviewer that *Y. pestis* can inject Yop effectors through its TTSS in different cell-types, and more specifically immune cells (PMNs, macrophages, dendritic cells, …). However, whether non-immune cells such as endothelial cells or fibroblasts are targeted *in vivo* remains mostly unknown. One study by Pechous *et al*, (PLoS PathoG, 2013) showed that in *Y. pestis* infected lungs, >96% of infected cells were CD45+ cells and that the 4% remaining infected cells were CD45- (endothelial/epithelial). We believe *Y. pestis* can, inject Yops effectors within any cell type it encounters *in vivo*. The studies published convincingly show that there is a specific bias toward immune phagocytic cells, probably demonstrating the efficient recruitment of these cells at the site of infection and the capacity of *Y. pestis* to counteract this immune response by injecting TTSS effectors within these cells. In the dNL, the wave of bacteria coming from the sinus and progressing through the cortex will meet any cell type, many PMNs, but also vascular cells, and probably inject Yop effectors in all cells.

The transmigration of PMNs through the vascular barrier, leading to the massive recruitment of immune cells in the parenchyma observed in the new Fig S1, probably leads to increased vascular permeability and maybe local bleeding. However, this would not induce complete physical degradation of the vessels themselves, such as the one observed in the *Y. pestis* infected dLN. The discussion has been amended to discuss this point according to the reviewer’s comment (l444-453)

Regarding the possibility that the phenotype observed might be due to intoxication, we believe the use of the B-lactamase reporter would not work in our “full-tissue” approach because of the need to incubate the individualized cells with the reporter.

2. The MOI used in the cell culture studies were extremely high. Justification for these numbers should be included. Better yet, a dose response might be more effective approach.

The MOI was chosen following preliminary results during the implementation of the project. Serially increasing MOI were used to infect HDMEC cells (1; 10; 50; 100; 1000) for various times (30’, 60’, 90, 120’, 240’). The result was always the same, with rounding of cells. But there is a MOI dose effect: MOI = 10 will take longer to reach the same % of rounding as MOI = 50 or 100. The MOI and the timing were chosen in order to reach a satisfying % of cell rounding without detaching or dying, thus allowing a reliable and amenable screening of mutants. The Material and Methods section has been amended to explain this methodology (l154-161).

3. Are the endothelial cells used in the in vitro studies polarized? Does this matter in the context of infection? Are the cells that round up targeted/injected by the T3SS?

We believe the cells are polarized since they adhere to the glass surface mimicking extracellular matrix underlying vessels and tight junctions are formed as the VE-Cadherin staining shows.

*Y. pestis* expresses several membrane proteins involved in adhesion to eukaryotic cells (Ail, Pla, Psa, YadBC). Some of the receptors for these “adhesins” are components of the extracellular matrix (Ail -> Fibronectin; Pla -> Laminin). One would expect the polarization might have an effect on the adhesion of the bacteria. However, we did not see any difference in the level of adhesion of *Y. pestis,* whether the cells were fully polarized (continuous monolayer with tight junctions) or incompletely polarized (no full junctions between cells). It seems that *Y. pestis* adhesion is a redundant function harbored by many proteins, thus limiting the impact of polarization in our model. Once the rounding is induced, the endothelial cells are no longer polarized.

Though there is no direct proof of Yops injection, all evidence points to the rounding of cells as a result of injection of Yops by TTSS. Black et al. showed a similar effect of *Y. pseudotuberculosis* YopE injection into HeLa cells (Black et al., Mol Mic, 2000). Supplemental figure Fig S4 shows that the mutant pYV- has no rounding effect, proving the role of the proteins encoded by the plasmid on the phenotype. If the bacteria are cultured at 28°C without pre-incubation at 37°C (necessary to induce the expression of the TTSS and Yop toxins), the rounding effect is delayed by 2-3hrs (time necessary to induce the expression of the TTSS; not shown). Supplemental Fig S3 shows that a lysate of bacteria cultured at 28°C or 37°C or the supernatant of bacteria cultured at both temperatures did not have such effect. In these experiments, the Yop effectors are certainly expressed at 37°C and put in contact with the membrane of the cells but without notable rounding effect. Thus, suggesting that the Yops have to be injected into the cell cytoplasm to display an effect.

4. Is the response by the endothelial cells due to the action of the Yop effectors or a host response to the infection (e.g., inflammasome activation and proptosis)? Infection with a strain expression the T3SS needle and lacking the effectors would help to understand the molecular mechanisms responsible for the phenotypes observed.

The rounding of endothelial cells can be the direct effect of Yop toxins, as a monoculture of HDMEC infected with *Y. pestis* shows rounding in the absence of any other partner (immune cells, or else). Future work will aim at deciphering the molecular determinant of the rounding and the mechanisms involved using several of deletion mutants and fused reporters with Yop secreting sequences.

5. Fig. 3: Not sure why both VE-cadherin and bacteria were both stained green. Is the increased colocalization of the green and red channels in 3B due to increased VE-cadherin at the cell membrane or are these bacteria? If bacteria, why different from 3D? The actin staining in CO92 pYV- (3D) also appears to be very different from all other samples. Is this a fair observation, or an artifact of the representative image shown?

We used two green stainings to visualize more objects within one experiment. We feel the double staining of junctions and bacteria is not problematic as both were easily distinguishable. Additionally, bacteria are also stained with DAPI, that lifts any doubt should there be.

The increased green staining appearing at the junction corresponds to an increased VE-Cadherin at the membrane. This is often seen when HDMEC cells are infected with *Y. pestis*. Note that the contrast of the image was slightly enhanced to better visualize the holes between cells, which might make the phenomenon appear stronger (It is now specified on the caption of the figure).

The actin staining in the panel 3D appears indeed different from other panels. This is because there is an increased number of actin stress cables in the cells infected with the pYV-cured mutant. The stress cables are induced under Rho GTPases activity, and it is known that some Yop effectors (e.g. YopE and YopT) inhibits some Rho GTPases activity. Thus, the increased number of stress cables may be explained by the infection of the cells with bacteria without the “relaxing” effect of the Yop effectors.

___________________________________________________________________________________________

**Editorial and Data Presentation Modifications?**
Use  this section for editorial suggestions as well as relatively minor modifications of existing data that would enhance clarity. If the only modifications needed are minor and/or editorial, you may wish to recommend “Minor Revision” or “Accept”.

**Reviewer #1**: Line 264-277: This section refers to a supplementary figure.  It seems like the figure could easily be included as regular figure in the text.
As recommended by the reviewer, former Fig S1 is now Fig 2.

Line 274: The authors refer to “secondary organs”, but I think they can specify spleen here. -> change made (l308).

Figure 2B: I think this figure would benefit from the addition of arrows pointing to the phenomena being described. -> Arrows added in the figure (now Fig 3B).

Line 308: The authors refer to “holes in cells”, but the holes seem to be gaps in the monolayer between cells at the junctions.  Thus, it is not clear how the “holes were observed in approximately 40% of the cells” was calculated

-> text corrected to “holes around cells” (l365). Cells were chosen randomly (using DAPI field), then the field was switched to Green and the presence of holes around the cell was enumerated. The method section has been modified accordingly (l202-204).

Figure 4A: It’s unclear why the Y-axis is in milligrams.  The methods implied that the fluorescence of FITC-dextran was what was being measured.  Is the fluorescence measurement being converted to mg?  If so, shouldn’t it be expressed as a concentration instead of just total mg?

Indeed, the fluorescence is what is measured, but the permeability of a barrier and the speed of diffusion through the barrier is expressed in terms of masses of molecules going through the barrier per unit of surface in a given time. It also depends on the molecular weight of the molecules. Here we use 4 kDa FITC-Dextran. The fluorescence of a diluted standard curve of FITC-Dex is measured for each experiment to convert fluorescence in mass. This allows evaluating the concentration of molecules in the lower chamber. The concentration was then converted to mass to allow the calculation of the speed of diffusion expressed in ug/min/cm^2^ (since the concentration also depends on the volume of medium in the lower chamber, the masse is more informative for the permeability). The principle has been added to the method section (l166-167).

Some suggestions for improving the text:

Thank you for these suggestions, the text has been corrected accordingly.

Line 33: change to “…remain poorly understood.” -> change made (l38).
Line 38: change to “…plasmid is responsible for…” -> change made (l43).
Line 45: “It is among the most important bacterial…” -> change made (l50).
Line 59: change to “Y. pestis can multiply within lymph nodes.  If the innate immune response in the lymph node is strong…” -> change (partly) made (l65-68).
Line 74: “…have been a striking feature…”-> change made (l81).
Line 84: “…factors that play…”-> change made (l91).
Line 89: “…activities capable of converting plasminogen to plasmin, thus degrading extracellular matrix and fibrin clots in vivo.”  -> change made (l96).
Line 94: “…associated with and essential for the virulence…” -> change made (l101).
Line 98: “…may be important during…”-> change made (l106).
Line 200: delete the sentence starting “This RFP CO92…”. Redundant-> sentence deleted.
Line 211: “…shows the highly vascularized medulla…” -> change made (l232).
Line 271: “…and the classical cfu…”-> change made (l305).
Line 272: “Contrary to the draining lymph node, colonized via the afferent lymph,” -> change made (l306).
Line 285: “…at this magnification we observed bacteria in the process of leaking out of the vessel into the lymph node parenchyma…”-> change made (l319).
Line 314: “…cells in either case…”-> change made (l370).
Line 315: “no toxins or bacterial…”-> change made (l371).
Line 316: “…host cell cytoplasm…”-> change made (l372).
Line 387: “…increased blood vessel permeability allows the crossing of bacteria, which could also explain systemic hemorrhages associated with plague.” -> change made (l466).
Line 392: “Y. pestis can breach…permeability that could provoke…”-> change made (l470).
Line 402: “…supernatant can produce…”-> change made (l481).

**Reviewer #2: Minor concerns/comments:**

Of note, pCD1 is the more common nomenclature for the Y. pestis plasmid as opposed to pYV which is typically used to denote the plasmind from Y. pseudotuberculosis or Y. enterocolitica.

The text has been amended and the name changed to pYV/pCD1 to be more generic. Other names of the pPla et pMT plasmids have been specified in the introduction as well.

The sentence beginning in 38 should likely read "pYV plasmid bears responsibility" or pYV plasmid is responsible"-> change made (l43).

Line 73- may want to slightly expand on what "bleedings" refers to specificlly (ie. internal hemorrhage?) -> change made (bleedings -> internal and external bleedings) (l80).

Lines 77-79- it is a little confusing as to how DIC (which is increased coagulation) could be responsible for hemhorage, may want to slightly clarify

DIC is characterized by an initial unregulated disseminated coagulation that eventually consumes essential factors of coagulation. A classic outcome of DIC is uncontrolled excessive bleeding.

Line 94- specify "inside the host cell cytosol"-> change made (l101)

Lines 94-96 state that the three plasmids are essential to virulence of the pathogenic Yersinia, but this is only true of Yersinia pestis- pCD1/pYV is the only plasmid of the 3 found in all three pathogenic Yersinia.

The reviewer is right. The text has been corrected: “These three plasmids are associated with *Y. pestis* virulence. The pYV/pCD1 plasmid is shared by and essential for the virulence of the three pathogenic Yersinia species” (l101-104).

Introduction may benefit from 1 or 2 sentences summarizing data or conculsions. -> A short summary of the data has been added to the introduction as requested (l109-115).

Line 187 italicize Y. pestis. -> correction made (l210).

Lines 190-191 should read "mechanisms involved....were never properly determined" or "mechanism involved...was never properly determined) -> correction made (l213-214).

The statement  in line 201 "retained the same virulence" does not have any confirmative data except for the LD50, and should perhaps read "retained the same LD50" (unless bacterial burden and survival is to be included in the supplement)- obviously a very minor concern if at all. -> Sentence removed.

Figure 1- Capsule appears to point to a follicle on the GFP panel, which may be confusing -> Fig 1 has been modified to lift the confusion.

It is difficult to determine if the images shown in Figure 1C are truly showing degraded cell remnants of vessels, as is suggested. Though this reviewer is not sure if it is possible, a control with an agent that disrupts the vasculature in some way or a marker of vascular damage would strengthen the argument.
This comment has been addressed in the response to reviewer#2 above.

On Page 12, it is indicated that bacteria are entering secondary infected lymph nodes through only the vasculature. Though likely, it is difficult to confirm that Figure 2B is showing "bacteria leaking to from the vessel into the parenchyma", it is not clear that those bacteria in the parenchyma originated in the vasculature vs entering the node in another manner (ie lymphatic system) and degrading vessels. This question isn't asked specifically (different timing, differential labeling, etc). Therefore language in this section with that regard should perhaps be tempered.

As explained in the text, the dense spots of bacteria are observed inside blood vessel lumens (Fig 3A, Fig S3) in the cortex of the lymph node, and distant from the subcapsular sinus. In these secondary LN, no bacteria are observable in the subcapsular sinus showing these LN are not infected through the lymph route at all. Therefore, the observation is consistent with a penetration of the bacteria in the lymph node via the bloodstream rather than via lymphatic flux. Note that these dense spots are very frequent in the secondary LN (often at least a dozen/LN).

The authors do  not comment on whether or not they see any disruption of the vasulature in the non-draining lymph nodes in which the bacteria have assumedly arrived by blood vessels. Is it proposed that the disruption of blood vessels strictly occurs from "outside" of the vessel?

The new supplemental figure (Fig S2C) presents a secondary LN at a more advanced stage of infection, in which the blood vasculature seems to disappear as in the dLN. We propose the same outcome for the LN whether the entry door is lymphatic or the blood circulation. The text has been amended to clarify the point (l322-325).

Supplementary Figure S2 is fairly striking, and might be included in the main text.

As recommended by the reviewer, former Fig S1 is now Fig 2.

In the discussion, it may be useful to comment on what it might mean if the vessel destruction is exclusive to bacteria that have arrived in the lymph nodes via the lymphatic system in the draining lymph node vs the destruction seen (or not seen) in the non-draining lymph node in which the bacteria have entered presumably via the vascular system.

We believe that the outcome will be the same for the LN, whether the entry door is lymphatic or the blood circulation. It has been clarified in the text as requested (l322-325).

**Reviewer #3: See minor comments below**.

Minor comments from below displaced:

Minor comments:
1. Line 29: Not sure what is meant by “a powerful pathogen”. Suggested rewording. -> It is a simple figure of speech used as hook. We like it but can remove it if the reviewer thinks it is inappropriate.

2. Yop effectors are not considered classical toxins. Suggest changing the use of “toxins” with “effectors”. -> The change has been made throughout the text.

3. Line 94: Only pCD1/pYV is conserved in all three Yersinia species, pPla and pMT are not in the enteric pathogens. Please revise tis sentence.

The text has been corrected: “These three plasmids are associated with *Y. pestis* virulence. The pYV/pCD1 plasmid is shared by and essential for the virulence of the three pathogenic Yersinia species” (l101-104).

4. The reader would benefit from including the structural references to Fig. 1B to orient them to the LN structure similar to shown in Fig. 1A.

Some structural identifications have been added to the figure. We also included the new figure (Fig S1) that shows the histopathological analysis of a dLN at D3 PI, at a level of infection close to the one in Fig 1. We believe the histopathological section helps to understand the structure of the Fig 1.

5. Line 273: I think the “lymphatic door” needs to clearly described – not sure this term is going to be widely understood. -> The sentence has been modified (l306).

6. Line 277: I think your conclusions would be better supported if you include the co-localization channel. Yellow color does not show up well on these images.

-> For some reason, PowerPoint seems to compensate the colors when images are included as Tiff format. The co-localization channel has been inserted as .jpg which appears to do the trick. The yellow color is better visualized now.

7. Line 349: You should include a description of the results of your positive control (mannitol) first then the Y. pestis data. -> The description has been added to the section (l408-413).

___________________________________________________________________________________________

**Summary and General Comments**
Use  this section to provide overall comments, discuss strengths/weaknesses of the study, novelty, significance, general execution and scholarship. You may also include additional comments for the author, including concerns about dual publication, research ethics, or publication ethics. If requesting major revision, please articulate the new experiments that are needed.

**Reviewer #1:** In their manuscript “The invasive pathogen Yersinia pestis disrupts host blood vasculature to spread and provoke hemorrhages” Mikaty et al. use in vivo and in vitro approaches to examine the effects of Y. pestis infection on the blood vessel endothelium.  For the in vivo work, the authors used a mouse strain expressing GFP in blood vessel endothelial cells, combined with red-fluorescent Y. pestis, to image bacteria and vessels in fixed draining lymph nodes, non-draining lymph nodes, and spleen.  For the in vitro work, the authors look at the effects of Y. pestis on the permeability of human endothelial cell monolayers.

Overall, the work is original and important, and the results are potentially very interesting.  However, I have concerns about some of the authors’ conclusions that I feel need to be addressed.  I also feel that the manuscript could use quite a bit of editing and I will suggest just some of the changes that could be made.

**Reviewer #2:** In this study, the authors utilize both in vivo and in vitro analysis to describe the pCD1-mediated degradation/destruction of the blood vessels in the primary draining lymph node after infection with Y. pestis. Using in vivo microscopy, they show presence of bacteria consistent with lymphatic vessel-mediate arrival in the draining lymph node, and clear disruption of blood vessels in the localized areas around the clusters of bacteria. The lack of co-localization between bacteria and vasculature in the draining lymph node is striking, as is the contrast between this finding and what is seen in the non-draining lymph node. It is proposed that this disruption/destruction of the vasculature is responsible for entry of the bacteria into the bloodstream and subsequent colonization of other lymphoid organs. The authors are able to show this in non-draining lymph nodes as well as the spleen. In the non-draining lymph nodes the authors see bacteria associated with the
vasculature as well as within the parenchyma of the lymph nodes.
   Overall, the manuscript is well-written and the data clearly presented. The microscopy is beautiful, and the authors do a nice job of piecing together the events that occur upon entry of Y. pestis into the draining lymph node, and the events subsequent to this critical step. This work most certainly contributes to our understanding of how Y. pestis disseminates within the body. The strengths of the manuscript are in the innovative microscopic technique, as well as in the comparison of colocalization of bacterial and vasculature within draining lymph nodes vs. non-draining lymph nodes and other organs. A weakness is that the manuscript is primarily compelling microscopy, which limits some of the conclusions. For instance, though the it is indicated that vessel destruction is observed as green blots within the staining, it can't be known for sure that this is what we are seeing, and though it is assumed that bacteria have leaked from he blood vasculature into the parenchyma
in the non-draining lymph nodes, this can't be confirmed for certain. It also might have been informative to have a sort of time course to observe draining and non-draining lymph nodes simultaneously at different time points (ie. earlier timepoints where we see small numbers of bacteria arriving at the draining lymph nodes vs. presumably no bacteria in the non-draining) to confirm the model proposed by the authors. Any weaknesses are offset by the fact that the microscopy in particular gives us clear insight into how Y. pestis behaves within the lymph nodes, and the authors do a great job of piecing together a narrative as to what is happening. AS a result, I consider the findings/observations to be highly significant to the field, and the manuscript paints a very compelling story, or at least lays the ground work for testing a model of dissemination.

**Reviewer #3: Summary**
Yersinia pestis is a highly invasive lymphotropic pathogen that must gain access to the blood in order to be effectively transmitted from the infected mammalian host to its insect vector. However, the mechanism(s) used by the bacterium to breach the lymph node to colonize the bloodstream are not clearly defined. Here Mikaty et al. describe a genetically modified mouse model, which expresses GFP primarily in endothelial cells, to visualize blood vasculature in the lymph nodes during bubonic plague. The authors use confocal microscopy to characterize the blood vessel architecture in the draining lymph node during Y. pestis infection, showing for the first time that blood vessels near Y. pestis appear to be damaged, possibly resulting in hemorrhaging. The authors also use primary human endothelial cells in cell culture to show that infection with Y. pestis results in what appears to be loss of tight junctions, and suggests that similar effects could occur in vivo, leading to
vessel damage and access to the blood stream by the bacterium. Finally, the authors demonstrate that the absence of the pCD1 virulence plasmid inhibits the ability of Y. pestis to induce morphological changes in endothelial cells and permeability of cell monolayers in vitro. This study introduces an exciting new in vivo model to help us to better understand the mechanisms used by Y. pestis to breach the lymph node and colonize the blood.

Comments:
This authors provide us with a clear characterization of the Flk-1GFP/+ mouse model in the context of bubonic plague, and implicates the Ysc T3SS and Yop effectors in causing endothelial damage and dissemination. While I think this model provides investigators with a powerful tool to study vascular damage during plague, the manuscript lacks clear mechanistic studies to justify some of the conclusions that the authors make in the manuscript. These conclusions could be significantly strengthened by additional experiments and/or a more detailed discussion of the results and data from previous published studies.

_____________________________________________________________________________________________________________


Figure Files:
While revising your submission, please upload your figure files to the Preflight Analysis and Conversion Engine (PACE) digital diagnostic tool, [https://urldefense.com/v3/__https://pacev2.apexcovantage.com__;!!JFdNOqOXpB6UZW0!6gjJHMzB1zWWNT8-uboY1UVto3an4_tbayzaiiJ5cm59BDv06RmVcusoKpo6Nf6-MzI721E$](https://urldefense.com/v3/__https:/pacev2.apexcovantage.com__;!!JFdNOqOXpB6UZW0!6gjJHMzB1zWWNT8-uboY1UVto3an4_tbayzaiiJ5cm59BDv06RmVcusoKpo6Nf6-MzI721E$) . PACE helps ensure that figures meet PLOS requirements. To use PACE, you must first register as a user. Then, login and navigate to the UPLOAD tab, where you will find detailed instructions on how to use the tool. If you encounter any issues or have any questions when using PACE, please email us at figures@plos.org.
 
Data Requirements:
Please note that, as a condition of publication, PLOS' data policy requires that you make available all data used to draw the conclusions outlined in your manuscript. Data must be deposited in an appropriate repository, included within the body of the manuscript, or uploaded as supporting information. This includes all numerical values that were used to generate graphs, histograms etc.. For an example see here: [https://urldefense.com/v3/__http://www.plosbiology.org/article/info*3Adoi*2F10.1371*2Fjournal.pbio.1001908*s5__;JSUlIw!!JFdNOqOXpB6UZW0!6gjJHMzB1zWWNT8-uboY1UVto3an4_tbayzaiiJ5cm59BDv06RmVcusoKpo6Nf6-0FyhGhc$](https://urldefense.com/v3/__http:/www.plosbiology.org/article/info*3Adoi*2F10.1371*2Fjournal.pbio.1001908*s5__;JSUlIw!!JFdNOqOXpB6UZW0!6gjJHMzB1zWWNT8-uboY1UVto3an4_tbayzaiiJ5cm59BDv06RmVcusoKpo6Nf6-0FyhGhc$) .
 
Reproducibility:
To enhance the reproducibility of your results, we recommend that you deposit your laboratory protocols in protocols.io, where a protocol can be assigned its own identifier (DOI) such that it can be cited independently in the future. Additionally, PLOS ONE offers an option to publish peer-reviewed clinical study protocols. Read more information on sharing protocols at [https://urldefense.com/v3/__https://plos.org/protocols?utm_medium=editorial-email&utm_source=authorletters&utm_campaign=protocols__;!!JFdNOqOXpB6UZW0!6gjJHMzB1zWWNT8-uboY1UVto3an4_tbayzaiiJ5cm59BDv06RmVcusoKpo6Nf6-yWrz6YM$](https://urldefense.com/v3/__https:/plos.org/protocols?utm_medium=editorial-email&utm_source=authorletters&utm_campaign=protocols__;!!JFdNOqOXpB6UZW0!6gjJHMzB1zWWNT8-uboY1UVto3an4_tbayzaiiJ5cm59BDv06RmVcusoKpo6Nf6-yWrz6YM$)

__________________________________________________
In compliance with data protection regulations, you may request that we remove your personal registration details at any time.  (Use the following URL: [https://urldefense.com/v3/__https://www.editorialmanager.com/pntd/login.asp?a=r__;!!JFdNOqOXpB6UZW0!6gjJHMzB1zWWNT8-uboY1UVto3an4_tbayzaiiJ5cm59BDv06RmVcusoKpo6Nf6-LXxwboI$](https://urldefense.com/v3/__https:/www.editorialmanager.com/pntd/login.asp?a=r__;!!JFdNOqOXpB6UZW0!6gjJHMzB1zWWNT8-uboY1UVto3an4_tbayzaiiJ5cm59BDv06RmVcusoKpo6Nf6-LXxwboI$) ). Please contact the publication office if you have any questions.
